# Supplementary material for: Adolescent, parent, and provider attitudes toward a machine learning based clinical decision support system for selecting treatment for youth depression
Source: BMC Med Inform Decis Mak. 2024 Jan 2;24:4. doi: 10.1186/s12911-023-02410-1 (PMC10759496; doi:10.1186/s12911-023-02410-1)
Supplement: Supplementary file 1 — Supplementary Material 1: CDSS-YD Parent/Teen Focus Group Guide [file 12911_2023_2410_MOESM1_ESM.docx]

**Supplementary Material 1: CDSS-YD Parent/Teen Focus Group Guide**

**Introduction**

1. Group introductions
2. Goals of the focus group

**Families’ Experiences with Treatment Planning**

One of the things we are hoping to understand is how using a computerized treatment planning guide might fit in with how families and treatment providers (therapists, doctors) make treatment decisions currently.

1. Can you share a little bit about how you and your (your child’s) treatment provider chose your (your child’s) treatment? What was that decision-making process like? How did you and the treatment provider decide whether to start therapy, medication, both, or some other treatment?
   1. How involved did you feel in the process of choosing your (your child’s) treatment? How did you feel about that level of involvement?
   2. What kinds of things were you thinking about when you decided what treatment to start?
   3. What do you think are the most important things in your mind when you think about the ideal treatment for your (your child’s) depression?
   4. Were there other people besides you, your parents/child, and your treatment provider, who influenced your decision (e.g., friends, people in your family, your community or religious group)? How did their opinions play a role in your decision making process to choose a treatment?
   5. Where there other options you considered to help with your (your child's) depression besides therapy, medication, or a combination? How did those ideas play a role in your decision making process to choose a treatment.
   6. Were there any aspects of the treatment planning process that you particularly liked or felt worked well for you?
   7. Were there any aspects of the treatment planning process that you felt did not work well for you?

**Overview of the CDSS-YD**

1. Give background on how the CDSS was developed
2. Screen share components of the CDSS-YD

**Feedback on the Questionnaires**

First, we’d like to get your feedback on the questionnaires. They take about 15 minutes (10 minutes) to complete. They can be completed it on a phone, tablet, or computer. It would be most helpful if they are completed prior to attending the appointment with the treatment provider, so that time in the appointment does not have to be used up filling out the questionnaires.

1. What do you think might get in the way of completing the questionnaires prior to the appointment?
2. What do you think would help make sure teens/parents complete the questionnaires prior to the appointment?
   1. *Probe for sending reminders by text or email, asking families to join appointment 15 minutes early to complete surveys.*

**Feedback on the CDSS-YD Treatment Recommendation Page**

I’m going to show you an example of the treatment recommendation page that is generated from the questionnaires completed by the parent, teen, and provider. The provider would share this treatment recommendation page with you and explain how it works. *[screen-share demo page. Explain each section.]*

1. Do you have any questions about the treatment recommendation page or how it would be used?
2. Now let’s look at each of the sections on this page. Looking at the treatment recommendation section (measures that inform the treatment recommendation section, additional measures section) –
   1. What do you think this section is saying?
   2. How clear or unclear is this section?
      1. What would make it clearer?
   3. How informative or uninformative is this section? Did you learn important information to help with your decision?
      1. What would make it more informative?
   4. How complete or incomplete is the information that you are provided?
      1. What other information would you like to have?
   5. How useful is this section for making a treatment decision?
      1. What would make it more useful?
3. What are your thoughts and feelings about your parent (your child) seeing the information on this page?
   1. What parts would you be comfortable with them seeing? What parts would you not be comfortable with them seeing?
      1. What led you to say that?
      2. Are there ways to make that more comfortable?
   2. Would you want to look at this with your provider privately or would you feel comfortable looking at it with your parent (your child) in the room too?
4. What additional suggestions do you have for how the treatment recommendation page can be made more useful or helpful?

**Feedback on the CDSS-YD Overall**

Now we’d like to get your feedback on the usefulness and potential impact of the decision guide overall - so, thinking about the parent questionnaires, the teen questionnaires, the provider-administered measures, and the treatment recommendation page.

1. How do you think using a decision guide like this would impact how much you feel involved in the treatment planning process?
   1. What would make you feel more involved?
2. To what extent do you think you would trust the treatment recommendation?
   1. What would make you trust it more?
3. How do you think using a decision guide like this would impact your confidence in the treatment recommendation that your provider gives you?
   1. What would make you feel more confident?
4. To what extent do you feel like using a decision guide like this would impact your relationship with your treatment provider?
   1. What would help improve your relationship with your treatment provider?
5. How do you think using a decision guide like this would impact your perception of your treatment provider’s knowledge or expertise?
   1. What would help you see using a tool like this as an indication that your provider is more knowledgeable?
6. How much do you think using a decision guide like this would help your (your child’s) depression get better?
   1. What would make it help your (your child’s) depression more?
7. What do you think we are missing with this decision guide?
8. Who are the people in your life (family, friends) or in your community or culture who would be important to talk with about this treatment guide a you are choosing your (your child’s) treatment? Are there people whose thumbs up would make you more confident that this is a helpful decision guide?
9. What do you think other teens (parents) would say about using a decision guide like this?
10. What do you think your parent (child) would say about using a decision guide like this?

**CDSS Provider Focus Group Guide**

**Introduction**

1. Group introductions
2. Goals of the focus group

**Providers’ Current Treatment Planning Practices**

One of the things we are hoping to understand is how the use of a computerized treatment planning tool might fit in with how providers are currently making decisions about the types of treatments that they recommend to families with a teen with depression.

1. Can you share a little bit about how you make decisions about what treatments you recommend to families when you are meeting with them?
   1. *What kinds of factors are you thinking about when you decide what to recommend? What might make you recommend therapy, or medication, or both?*
   2. *Are there any aspects of your evaluation and treatment planning process that you feel are not working well for you right now? Times when you feel stuck or treatment planning feels tricky?*
   3. *At what point in your usual clinical workflow do you usually feel like you’re ready to make a treatment recommendation?*

**Overview of the CDSS-YD**

1. Give background on how the CDSS was developed
2. Screen share components of the CDSS-YD

**Feedback on the CDSS Treatment Recommendation Page**

We’re going to email you an example of the treatment recommendation page that is generated from the measures completed by the parent, teen, and provider. You can navigate through it, and we’d like to talk about the usefulness of the page. *[Email demo page]*

1. As you are looking through the treatment recommendation page. I want you to jot down: Where do your eyes go first? What information did you take in? Where do your eyes go next? What information did you take in? And so on.
   1. *What drew your attention there?*
   2. *Was it intuitive what you were looking at?*
2. Does you have any questions about the tool or how it would be used?
3. Now let’s look at each of the sections on this page *[screen-share demo page]*. Looking at the treatment recommendation section (measures that inform the treatment recommendation section, additional measures section), how useful is this section for making a treatment recommendation?
   - *What would make it more useful?*
   - *Of these sections, which one(s) would you actually look at? How would you use the information in that section?*
4. What additional suggestions do you have for how the treatment recommendation page can be made more useful in practice?

**Feedback on the CDSS Tool Overall**

[*Questions in blue are to be asked if time allows*]

Now we’d like to get your feedback on the usefulness and potential impact of the tool overall - so, thinking about the parent survey, the teen survey, the provider-administered measures, and the treatment recommendation page.

1. What would make this tool more usable/user-friendly in practice?
2. Next, I’d like to ask about the extent to which you think you would trust the treatment recommendation.
   1. *What would make you trust it more?*
3. To what extent do you feel like using a tool like this would impact your therapeutic relationship with the family?
   1. *What would help improve the therapeutic relationship?*
4. How much do you think using a tool like this would affect patients’ treatment outcomes?
   1. *What would make it improve outcomes more?*
5. What do you think your co-workers would say about the tool?
6. What are we missing with this tool?
7. What would make this more beneficial to the care you provide?

**Feedback on CDSS Implementation**

[*Questions in blue are to be asked if time allows*]

Now, we’d like you think about actually using this tool yourself when meeting with a family to discuss treatment planning.

1. How could you see this tool fitting into your practice?
   1. *Where would you position this within your usual clinical workflow with patients?*
2. What would make this something you’d **want** to use?
3. What would make you more likely to actually use this?
   1. *What are the most important considerations for ensuring providers use this?*
   2. *If not mentioned: what considerations regarding leadership and messaging would make you more likely to use this tool? Considerations within your workflow (e.g., EHR, reminders, supervision)? Considerations in your clinic? or within Fairview?*
4. What do you see as the keys to setting providers up for success in the treatment recommendation process?
5. What would get people excited about using this?
6. What kinds of additional questions should we be asking of providers as we continue to learn more about how to make this tool better?
