# Supplementary material for: Adolescent, parent, and provider attitudes toward a machine learning based clinical decision support system for selecting treatment for youth depression
Source: BMC Med Inform Decis Mak. 2024 Jan 2;24:4. doi: 10.1186/s12911-023-02410-1 (PMC10759496; doi:10.1186/s12911-023-02410-1)
Supplement: Supplementary file 2 — Supplementary Material 2: CDSS Treatment Selection [file 12911_2023_2410_MOESM2_ESM.docx]

Supplementary Material 2: CDSS Treatment Selection

1. Which treatment did you recommend?
   1. CBT
   2. Medication
   3. Combination Treatment
   4. Other: __________________________
2. Why did you recommend this treatment? _______________________
3. Which treatment did the adolescent prefer?
   1. CBT
   2. Medication
   3. Combination Treatment
   4. Other: __________________________
4. Why did the adolescent prefer this treatment? __________________________
5. Which treatment did the parent/caregiver(s) prefer?
   1. CBT
   2. Medication
   3. Combination Treatment
   4. Other: __________________________
6. Why did the parent/caregiver(s) prefer this treatment? __________________________
7. Which treatment approach did the family select?
   1. CBT
   2. Medication
   3. Combination Treatment
   4. Other: __________________________
8. Why did the family select this treatment? ________________________
9. Additional comments about the treatment selection process:
10. What did you like about using the CDSS with this family? What worked well?
11. What did you not like about using the CDSS with this family? What didn’t work well?
